# Emotion-Driven Analysis and Control of Human-Robot Interactions in Collaborative Applications

**DOI:** 10.3390/s21144626

**Published:** 2021-07-06

**Authors:** Aitor Toichoa Eyam, Wael M. Mohammed, Jose L. Martinez Lastra

**Affiliations:** FAST-Lab, Faculty of Engineering and Natural Sciences, Tampere University, 3320 Tampere, Finland; aitor.toichoaeyam@gmail.com (A.T.E.); jose.martinezlastra@tuni.fi (J.L.M.L.)

**Keywords:** human-robot interaction, human-robot collaboration, trust, emotions, emotion-driven, electroencephalography (EEG), human-in-the-loop, adaptive systems, industrial applications, human-machine interface, emotion recognition, collaborative robots, cobot

## Abstract

The utilization of robotic systems has been increasing in the last decade. This increase has been derived by the evolvement in the computational capabilities, communication systems, and the information systems of the manufacturing systems which is reflected in the concept of Industry 4.0. Furthermore, the robotics systems are continuously required to address new challenges in the industrial and manufacturing domain, like keeping humans in the loop, among other challenges. Briefly, the keeping humans in the loop concept focuses on closing the gap between humans and machines by introducing a safe and trustworthy environment for the human workers to work side by side with robots and machines. It aims at increasing the engagement of the human as the automation level increases rather than replacing the human, which can be nearly impossible in some applications. Consequently, the collaborative robots (Cobots) have been created to allow physical interaction with the human worker. However, these cobots still lack of recognizing the human emotional state. In this regard, this paper presents an approach for adapting cobot parameters to the emotional state of the human worker. The approach utilizes the Electroencephalography (EEG) technology for digitizing and understanding the human emotional state. Afterwards, the parameters of the cobot are instantly adjusted to keep the human emotional state in a desirable range which increases the confidence and the trust between the human and the cobot. In addition, the paper includes a review on technologies and methods for emotional sensing and recognition. Finally, this approach is tested on an ABB YuMi cobot with commercially available EEG headset.

## 1. Introduction

The concept of Industry 4.0, which was introduced by the German government in 2011, has been slowly but steadily adapted by the manufacturing systems’ vendors [1,2]. Industry 4.0 relies on technologies such as the Industrial Internet of Things (IIoT) [3] and Industrial Cyber-Physical Systems (ICPS) [4], where both the physical and virtual worlds are linked in order to maximize the benefits of the advances in the Information and Communication Technology (ICT) [1,5]. Despite that the Industry 4.0 concept has not reach its maximum potential of development, and as the technology is evolving with an exponential pattern, some researches have started defining the concept of Industry 5.0 [6,7,8]. In this regard, several authors prognosticate that Industry 5.0 will focus on keeping humans in the loop of processes, creating synergies between humans and machines [7,9]. Furthermore, the late advances in the robotics domain introduced the concept of Human–Robot Collaboration (HRC) and Human-Robot Interaction (HRI) as potential research and innovation disciplines [10,11]. In fact, these concepts are highly dependent on the trust and the understanding of human cognitive behavior while working with robots as depicted in [12].

The use of HRC and HRI technologies in industrial applications creates comfortable working atmosphere for employees, which improves their trust in machines and working performance [13]. Some of these applications, as in [14], permits machines to adapt to the human employee instead of the human who adapts to the machine. This approach is defined as Adaptive Automation System (AAS) in [10]. These are systems that adjust their states and characteristics depending on the operational context [15]. As technology evolves, more robots are employed in industries while sharing working areas with humans and acting as assistants on daily routines [16,17]. In order to fulfil their duties in the best possible way, robots must adjust their parameters to the user needs. With regards to human safety, industrial robotics’ vendors develop new types of robots known as “Collaborative Robots” (Cobots) [18,19]. Cobots are created to work hand-by-hand with humans, without the need of physical separation using fences and, consequently, without endangering the human [20]. These robots are designed with additional features like sensitive collision detection that allows avoiding the physical harm to the nearby human. However, the emotional and behavioral recognition of the human is yet to be developed, which in return allows these robots to collaborate seamlessly, increasing the safety and comfort of the human.

Several emotion-recognition methodologies, techniques and technologies are developed and used for understanding the human emotions. An example of technology that is becoming more relevant in this field is electroencephalography (EEG) [21]. EEG is a non-invasive method that allows for detecting the brain activity of a person. One of the reasons why EEG is being used to interpret emotional patterns is that brain activity is an internal signal, meaning that is harder to influence voluntarily, giving a more realistic result of the emotion interpretation [22]. With the goal of applying EEG technology outside the medical domain, there are some companies that are producing EEG headsets that can be used for industrial applications as robot manipulation [23], vehicle (drone) control [24], or even video games [25,26]. 

According to [27], mental states of the workers as boredom, fatigue and stress are one of the main causes of productivity reduction and labor accidents in factories. Thus, this paper aims at proposing a solution for adjusting the interactions between the human workers and the cobots based on the human mental and emotional states. In more details, the objectives of this paper include:

- Conducting through literature review on the technologies and the utilization of the EEG sensors in industrial applications.
- Investigating the usage of EEG sensors for measuring the emotions of the human.
- Designing, architecting, and developing a system for analyzing and controlling cobot’s parameters based on the human emotions.
- Conducting experiments to validate the proposed system.

The structure of this article is composed of six sections. Section 1 presents the main aspects and ideas that are behind this paper. Section 2 shows the state of art used as the foundation for the development of this investigation. Section 3 presents the proposed scenario and the selection of techniques and technologies needed to achieve the objectives of the research. Section 4 focuses on the implementation of the proposal. And finally, Section 5 presents an analysis of the implementation, conclusions, and future work.

## 2. State of the Art

### 2.1. Human-Robot Interactions and Collaboration

The adaptation of human presence in the industrial environments has been continually researched and developed in last decades. The target has been always providing safe, easy-to-use, simple, and reliable interfaces between humans and the factory shop floor machines [14]. The importance of such objective includes building trustworthy and ergonomically comfortable surroundings for the human workers. Additionally, and with the advances in the manufacturing and industrial systems, several machines and robots are now cable of collaborating with the human workers. This means human and robots work together simultaneously. This collaboration creates additional challenges for the system designers and integrators to assure the aforementioned objectives. Hancock et al. in [28] presented a study on the trustworthy between robots and human. The paper concluded that the dominant factor of human trust in robots during operations is the overall operational performance of the robot, like speed and acceleration. The physical appearance and the size contribution are labelled as moderate. While the human factor effect appears to be marginal. Moreover, Sanders et al. in [29] depicts an updated model for addressing the HRC called as Human Robot Team Trust (HRTT). On one hand, the HRTT considers the human as a resource with certain abilities that with training, the resource can gain or lose trust. On the other hand, it realizes the robots as resources with known performance attributes and can be included as needed in the design phase of the collaborative process.

Another aspect of the HRI is the safety and ergonomics of the worker. In this regard, humans are highly flexible resources due to the high degree of motion of the physical structure and high level of intelligence and recognition. Additionally, the high level of adaptability of human contribute to the flexibility of the collaborative work with robots which keeps the human at the factory shopfloor even in a highly automated environment [30]. Nonetheless, working in collaboration with machines and robots requires taking addition safety measures. This can be seen in the addition of fences and monitoring systems for safety that are necessitated at the factory shopfloor. However, the new concepts of collaboration require open spaces and fenceless machines and robots which contradicts with the traditional safety measures in the majority of the today’s factories. For instance, Olesya Ogorodnikova in [31] presents some approaches for risk estimation and management in HRI and HRC applications. In the presented paper, the author discusses the lack of industry-specific standards that are needed to support safety and health measures in the factories for HRI and HRC applications. Even though the International Organization for Standardization (ISO) published several documents like ISO-12100 and ISO-10218 among many for addressing the safety of robots and machines, the HRC and HRI are still not fully adapted in industrial scenarios. Therefore, the human and machine collaboration is still under development in terms of safety. 

In relation to robotics, Artificial Intelligence (AI) based applications can be introduced for teaching the robot on how to predict and act in the presence of the human. As an example, and in a recent publication, Ionescu in [32] demonstrates the usage of the concept of reinforcement learning in an Adaptive Simplex Architecture (ASA) approach for collision avoidance with human. Furthermore, the implementations of new technologies in vision systems using high speed cameras and 3D cameras can enhance the safety of the human worker as depicted in [33,34]. Furthermore, the use of cloud services like Amazon Web Services (AWS) can simplify the integration of AI-based systems in the domain of HMI and HRC. As an example, Olatz et al. in [14] demonstrates the use of the Amazon’s deep lens camera for detecting the human and adjusting the robot operations based on the worker profile.

### 2.2. Human Emotions: Sensing and Analyzing

In the search for learning and understanding the interpretation of the human’s emotions, researchers have developed two common methodologies for classifying emotions. The first method consists of differentiating a specific set of basic emotions. Similar to the primary colors, the combination of these emotions results in new emotional states. Plutchik in [35] exposes an example of this technique defining eight primary emotions: terror, rage, ecstasy, grief, trust, loathing, anticipation and astonishment. On the other hand, the second approach proposes classifying the emotions by using a dimensional space. Contingent upon their axis’s values, each mental state will occupy a different position in space. J. A. Russell presented this methodology using a bidimensional space delineated by valence as abscissas and arousal as ordinate [36]. This approach encounters a problem when it must classify mental states which are akin to each other such as stress, anger or fear. In this type of situation, a bipolar space is not enough to distinguish the emotions. With the purpose of solving this issue, the system was improved by adding a third axe, giving rise to a three-dimensional space delimited by the axes Pleasure-Arousal-Dominance (PAD) [37]. The first two axes, Pleasure and Arousal represent the Valence-Arousal plane, being Dominance the dimension that helps to solve the problem of states with similar Valence-Arousal values.

Taking into consideration both patterns of classification, the dimensional approach is used with more frequency than the basic-emotions method due to its practicality, versatility and facility to differentiate emotions. In order to detect the mental states of a person, there have been developed several techniques to detect mental states in human beings. Some of these techniques are used on HCI devices. Firstly, emotions are recognized and then classified using the abovementioned methods. The most commonly used procedures of recognition are:

- **Facial recognition**, being one of the most popular methods among emotion recognition, focuses on analyzing the movements of the 43 muscles of the face. By the combination of moving these muscles, humans express their emotions unconsciously with a facial expression as an output. This recognition technique is based on the basic emotions approach which is illustrated in [36]. This approach considers devices using facial recognition that tracks variations in the muscles of the face. Usually, the main targets are eyebrows, eyes, nose, and mouth movements. The articulation of these parts of the face creates a map of points which is compared with a database [36,38,39]. This type of emotions’ recognition can be inaccurate. In this regard, research shows that depending on the cultural background of a person, reaction to the same stimulus might differ [40]. Also, it is possible to counterfeit facial expressions, as, for instance, the professional poker player tends to do.
- **Speech recognition** is based on the analysis of common patterns in the human voice while speaking. The verbal communication has changed the capacity of humans to exchange information, generate interactions and express ideas or emotions. Therefore, speech analysis is a great tool for extracting data related to mental state. In order to properly study this field of communication, it is necessary to take into consideration several features such as pitch, energy, resonance, frequency, vocabulary, age, sex, etc. [41]. Most of the approaches employing speech recognition use resonance, pitch and energy to determine the arousal level on the dimensional methodology. Alternatively, linguistic features as lexis, grammar or fluency are used to classify emotions in the valence dimension [42]. After the analysis, it is necessary to use a database to categorize the mental states. Furthermore, devices based on speech recognition find controversy while determining the arousal levels of emotions, and it requires an accurate database. Like facial recognition, speech recognition can be altered.
- **Body language** is defined as unconscious human body movements that create understandable postures and gestures. It is the most widespread and subtle form of communication used by human beings. According to [43], 65% of the humans’ non-verbal communication is achieved by body language. Commonly, technologies using body language to recognize mental states by utilizing visual recognition algorithms to differentiate between body postures, gender and age [44]. The recognition based on the body language can be considered inaccurate due to different cultural habits between people.
- **Electrocardiography (ECG)** is an examination method that presents the electrical activity of the human heart [45]. It is accomplished by using machines that comprise of electrodes and central recoding unit that records the electrical signals produced by each pulse of the heart. These electrodes are positioned at well-known locations on the chest with the aim of capturing heartbeat signals. Afterwards, some techniques can be applied to detect emotions. After analysis, dimensional methodology with Valence-Arousal dimensions is used to classify mental states [46]. The most beneficial aspect that has ECG over the previous emotion recognition techniques is that it is really difficult for people to voluntarily influence their heartbeats, making it more difficult to hide emotions from detection.
- **Electroencephalography (EEG)**: Similar to ECG, EEG is a non-invasive neurophysiological exploration technique that tracks the electrical signals generated by the brain. The bioelectrical activity of the brain can be measured by setting a group of electrodes on predetermined locations from an electroencephalograph device on the scalp. Usually, the electrodes are set following the 10–20 system [47]. Each electrode registers the voltage produced by the neuronal activity at the site where it is placed. Depending on the frequency of the sensed signals, five types of brainwaves can be defined: delta, theta, alpha, beta and gamma [48].
  ○ Delta waves (from 0.5 to 4 Hz): Associated with dreamless state of the sleep.
  ○ Theta waves (from 4 to 8 Hz): Usually generated in REM phase, deep meditation, or flow state.
  ○ Alpha waves (from 8 to 12 Hz): This is the most common brain rhythm as it is produced in an awake state while being concentrated and relaxed.
  ○ Beta waves (from 12 to 25 Hz): It use to be categorized into subtypes. These subtypes include Low beta (from 12 to 15 Hz) which is associated with idle state. Beta (from 15 to 23 Hz) that is related to high attention, engagement, or task-oriented work. Finally, High beta (from 23 to 38 Hz) which is linked with stress, complex analysis, or new experiences.
  ○ Gamma waves (from 25, 45 Hz): Produced while processing information, making voluntary movements, multi-tasking or in moments of great inspiration.

By combining the signals that are generated by the brain with the location on the scalp, an AI-based application can determine the state of emotions that the individual experiences. In this regard, the human brain is divided into four Lobes: frontal, parietal, temporal and occipital [49]. Following the signals generated by each lobe, human emotions can be categorized and identified. Researchers have confirmed this theory by relating brainwaves with emotions and classifying them using the Pleasure-Arousal-Dominance (PAD) model. The Pleasure dimension is related to the hemisphere where alpha wave appears [49,50,51,52]. In addition, the beta wave rhythm relates to an alert state of the individual, while the alpha wave relates to the relaxed condition. The ratio of beta to alpha brainwaves defines the Arousal dimension [37,50,51]. Lastly, the Dominance is determined by the increment of beta to alpha ratio in the frontal lobe and an increase of beta waves at the parietal lobe [37,50,51,52]. These complicated mechanisms make devices using EEG for emotion recognition almost impossible to be deceived in purpose by the subject individual.

In order to monitor mental activity and emotions using EEG, it is necessary to have an EEG headset. There have been various headsets developed that use the 10–20 system, registering the needed information from the lobes allowing emotions analysis and recognition. Some of the most relevant commercially available headsets include:

- Neurosky [53]: This headset has a unique EEG dry electrode positioned on the FP1 of the 10–20 system. The company also offers software for brainwaves analysis.
- OpenBCI [54]: Offers 3D printed headsets with 35 different positions for setting the electrodes in the 10–20 system. The dedicated software allows monitoring 16 EEG channels and registering raw data.
- Emotiv [55]: The company presents 4 main headsets: Insight, Epoc+, EpocX and Epoc Flex, each headset with more available electrodes positions than the previous one. 5 dry electrodes for the Insight model, 14 EEG channels with saline electrodes for both Epoc+ and EpocX, and 34 sensors for the Epoc Flex. The main characteristic of these headsets is that Emotiv’s software includes emotional detection, mental commands training and monitoring raw data.
- Cognionics [56]: It has a great catalogue of headsets from 8 to 128 channels with dry or saline electrodes. In addition to its broad headset selection, it is possible to acquire data with its software and develop applications using several programming languages.

As a reflection of the importance that is earning emotion recognition for HRI, more emotion-driven applications have been developed. Some of the most relevant examples that can be found nowadays are:

- Pepper [57]: Humanoid robot developed by SoftBank Robotics, it can recognize basic human mental states and having interactions with people. Usually used for educational purposes, giving guidance or as reception assistance.
- NAO [57]: Also created by SoftBank Robotics, the main characteristic of this robot is that it can be programmed. As a consequence, it is mainly used for education goals, healthcare, and entertainment.
- Roboy [58]: A robot generated by the University of Zurich which tries to mimic human behavior with the aim of improving HRI. Roboy can understand users, perform conversations, and give emotional feedback for a received stimulus.
- Affectiva [59]: This company develops applications based on detection, measurement and analysis of mental states. The company achieves answering emotion stimulus by using artificial intelligence algorithms, verbal and non-verbal analysis, facial recognition combined with large databases.

In relation with studying HRI using humans’ brain signals, Maryam et al. in [60] presents a deep analysis in the future potential of using EEG analysis for enhancing human-robot interactions. In the presented paper, the authors list the advantages of such an approach: providing a socially engaging training for the users, decreasing the time for calibration and configuration of the robots, and providing systematically viable solution for users’ performance assessment while working with robots. In addition, Matteo et al. in [61] highlights the challenge of using EEG in human-robot interactions environment which are the readiness of the technology to provide a reliable solution for measuring and understanding correctly the human emotions.

## 3. Proposal and Technology Selection

The main aim of this research is improving the HRI implementation by permitting cobots to adapt to the emotional states of the human worker. To fulfil this goal, it is necessary to understand human emotions. Thus, the first activity is sensing and recognizing human emotions and feelings. Secondly, the approach must correlate the changes in emotions and feelings with the collaborative process tasks and activities. Thirdly, the robot is required to react to the changes in the human emotions to improve the worker experience. Finally, the approach needs to provide feedback to the user about the robot adaptation based on the changes of the emotions for the training purposes.

### 3.1. Recognition of the Human’s Emotions

For understanding and digitizing the emotional state of the human worker, the emotion detection techniques that are mentioned in the previous section (Section 2.2) might provide a solution. The facial, speech and body recognition can be useful for estimating the state of the worker. Nonetheless, these methods do not provide an accurate enough estimation of the real emotional state of the human. As the application focuses on safety measures, these methods can increase the risk of misunderstanding the human, which can lead to an unsafe HRC experience. Therefore, the user will not be establishing a conversation with the cobot and the working positions will be similar in each assembly. Moreover, facial recognition is not the most appropriate tool for this case as workers faces can be obscured while working. As a result, in this case, only ECG and EEG could be considered as powerful techniques for certain measurements. Comparing the ECG and the EEG regarding the *PAD* approach, ECG data well represents the arousal dimension, but is not much accurate with the Pleasure dimension. On the contrary, EEG achieves optimal results in both Pleasure and Arousal dimensions [39]. Therefore, and as the mental state could suffer alterations in more than one axe of the *PAD* space, it seems optimal to select EEG as an emotion detection technique.

**Figure 1:**
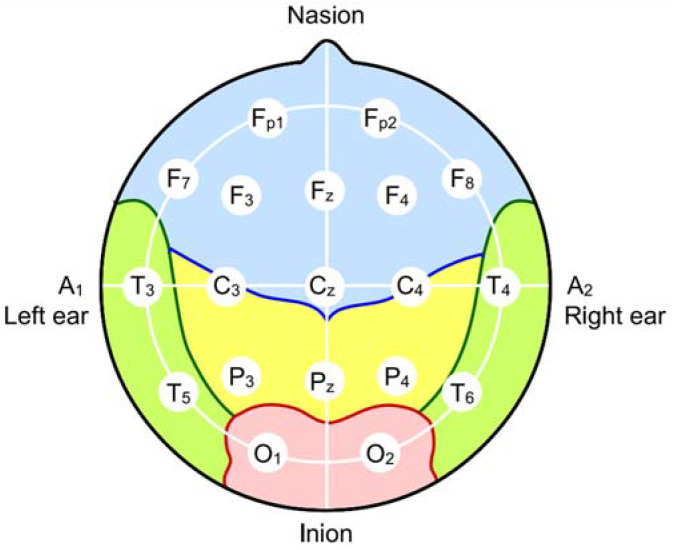
Placement of the electrodes using 10–20 system [49].

The EEG follows a well-defined placement of the electrodes as depicted in Figure 1. This system is known as the 10–20 system which refers to the percentage of the distancing of the electrodes. Once the electrodes are placed correctly, the sensors will convert the brain activities into signals with different frequencies. As described in the previous Section 2.2, each frequency is associated with a state which can reflect the status of the brain. With the combination of the electrodes’ locations with respect to the lobes and the frequency that is generated form the electrodes, the PAD space can be created. The PAD space will show the emotions based on the values of pleasure, arousal and dominance. Finally, the PAD space signals can be converted into several classes of emotions as depicted in [62,63]. These classifiers are usually provided with the headset that includes the electrodes. As an example, Table 1 presents the emotions that Epoc+ headset recognize.

### 3.2. System Architecture and Interactions

**Figure 2:**
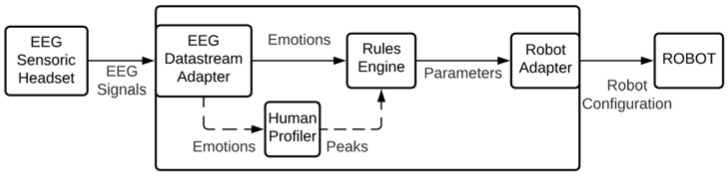
System architecture for human emotion-driven robot configurator.

The concept of continuously adapting the robot’s parameters to human emotions requires real-time data analysis. This means the system needs to receive the data stream from the headset and provide the needed analysis with minimum latency. As depicted in Figure 2, the approach considers a rule-based model for adapting the robot parameters. The advantage of such approach includes the simplicity of programming the rule or using rule-based reasoning engine for the robot adaptation. On the contrary, fine tuning of the system requires revision of all rules which might requires extra effort.

The data generated form the EEG headset is received by the EEG Datastream Adapter. This adapter performs the conversion form EEG signals to human emotions as described in Section 3.1. The detected emotions pass to two components Human Profiler and Rules Engine. It is used for calibrating the system for the user emotions for gaining maximum performance. In more detail, the human reaction to a certain stimulus is different. Therefore, the generated emotions levels will be different. The Human Profiler is only used during the configuration of the system. Thus, it is represented in dotted arrows in the figure. The output of the profiler is fed to the Rules Engine. The rules engine is the component that decides the parameters of the robot based on the emotions and the human profile. The rules are constructed following the first order logic. Finally, the robot adapter is the component that translates the rules conclusion into parameters for the robot. This component is preconfigured with set of parameters that can be modified based on the process and the robot type.

**Figure 3:**
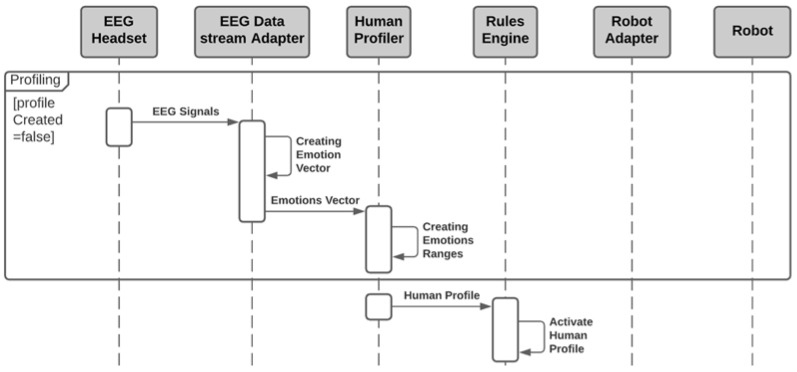
Sequence diagram of the human profiling process.

The profiling process is needed for understanding the reaction of each user where the intensity of the emotion can be accurately measured. The human profiling sequence is depicted in Figure 3. As shown in the figure, the sequence starts by reading the electrodes EEG signals. This signal is received by the EEG Datastream Adapter. This adapter will create the emotions vector. This vector will be created in a periodic manner based on the EEG signals and the extracted emotions. For instance, some emotion requires analysis of EEG signals for a certain period like the relaxation and the long-term excitement. Afterwards, the emotions vector is passed to the human profiler where the calculation of the emotions ranges is conducted. This process includes exposing the possible users to the similar stimulus, then recording the reaction of every user to determine the peaks in the emotions. The stimulus may include several clips of videos, several pieces of music or actual and virtual experiment. Finally, the ranges of the extracted emotions for each user can be stored by the Rules Engine for adapting the robot parameters during the operational mode. 

**Figure 4:**
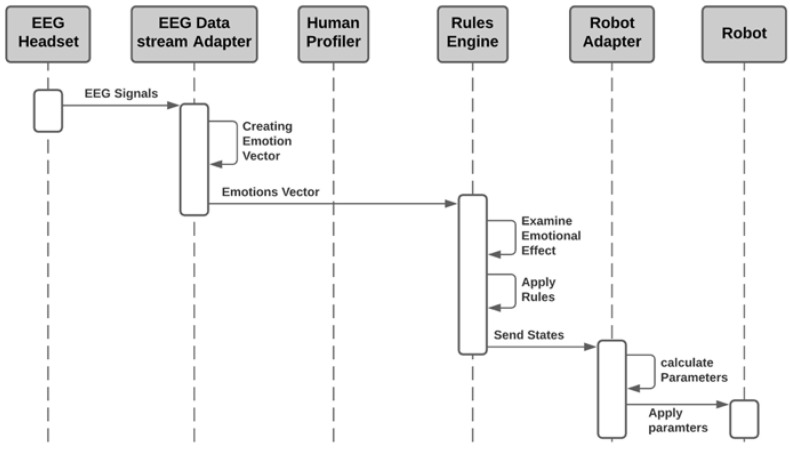
Sequence diagram of the operational mode.

Once the profiling process is finished, the system can be put in the operational mode. The sequence of the operational mode starts by receiving the EEG signals in the EEG Data Stream Adapter as depicted in Figure 4. Like the human profiling mode, the operational mode includes converting the EEG signals to emotions vector. This vector is fed to the Rules Engines. The Rules Engine is configured with predefined first order rules and calibrated to human reactions. This allows the Rules Engine to provide the accurate assessment that the robot is required to perform to adapt to the human emotions. Finally, the Robot Adapter communicate with the robot for reconfiguring the robot’s parameters according to the human’s emotions and feelings.

## 4. Implementation

### 4.1. The Use Case

**Figure 5:**
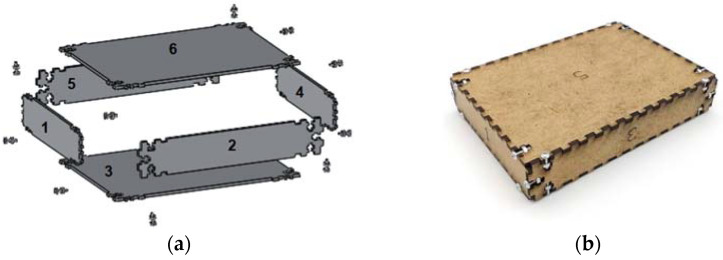
The object that is used for assembly process [64]. On the right (**a**) the box parts and on the left (**b**) fully assembled box.

In order to demonstrate the presented approach in the previous section, the use case needs to be collaborative and encapsulates characteristics that could be extrapolated to several situations in the industrial field. It is important to emphasize the collaboration concept where human and robots perform tasks simultaneously. In addition, the use case needs to be repeatable with very minor changes. This requirement is important for assessing the human emotion with several identical repetitions. Some suitable use cases can be assembling of rigid parts with very clear steps. In fact, assembly is a process quite spread in the industrial field, there are different methods of assembly and it also could be performed collaboratively. As the task should be simple but representative, the proposed item to be assembled is a small wooden box. In order to assemble the box, the robot and the user should join the six sides that compose the box and join them with screws and nuts as show in Figure 5a. Synchronizing their steps, the robot will fixate the box sides in position while the human will be preforming the screwing tasks. Figure 5b presents a fully assembled box using the collaborative use case.

**Figure 6:**
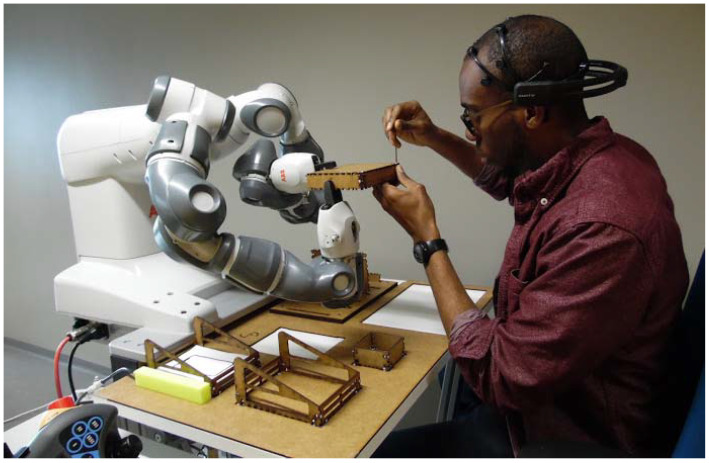
Layout of the workstation for the assembly process.

This use case involves the usage of a dual arm collaborative robot. More precisely, and as depicted in Figure 6, the used robot the ABB IRB14000 which is also known as YuMi cobot. As seen in the figure, the human sets opposite to the robot and both the robot and the human worker perform the tasks together. The assembly process of the box lasts for 5 min approximately. It requires critical abilities such as precision, accuracy, coordination and synchronization. 

**Figure 7:**
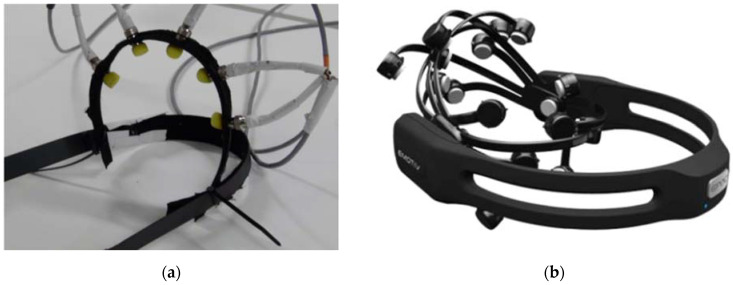
The used EEG headsets for this research: (**a**) in-house developed 5-electrodes EEG headset using the OpenEEG project; (**b**) the Epoc+ headset with 14 electrodes by Emotiv.

For the EEG sensing headset, initially, the aim was to develop a headset with several electrodes in-house using the OpenEEG project in [65]. The approach was to develop a 5-electrodes set and test the signals that are generated from the headset. The objective of such aim is allowing the researchers to increase the number of the electrodes as needed which enhances the quality of the overall emotions’ detections. Furthermore, it will provide flexibility in the placement of the electrodes so several approaches can be experimented. Besides the in-house set, the authors used the headset Epoc+ from Emotiv to compare the signals of the EEG between the two sets. Figure 7a shows the in-house developed set with 5 electrodes and Figure 7b shows the Epoc+ headset. The Epoc+ includes 14 electrodes and is equipped with Bluetooth connection for wireless communication which is very appreciated. In addition, the Epoc+ is supplied with the proper software that allows simple and straightforward recognition of the emotions. After the initial tests of the in-house headset, the results showed a very high noise to signal ratio which caused poor accuracy for of the detection of the emotions. This high ratio is mainly caused by the quality of the electrodes themselves, the impedance of the electronics and the connecting wires, and the amount pressure that the electrodes exserted on the scalp. For the electrodes and the electronics, the researchers followed exactly the recommendation in the OpenEEG project which leaves no room for correction. For the wiring, the researchers tried to use different types of cables including co-axial cables and twisted pairs with different gauges and parameters, but the overall improvement was not noticeable. However, a noticeable improvement have been noted once an external pressure pushed the electrodes on the scalp. The challenge was to build, or 3D print the housing so that all electrodes applies an equal amount of pressure. This equality of the pressure was needed as the developed application showed low repeatability in the readings. This problem was not easy to solve in and it requires deeper investigation of holding the electrodes accurately and properly. In addition to the aforementioned problems, the heavy weight and bulkiness of the inhouse developed headset led to discarding this option and only utilise the Epoc+ headset.

**Figure 8:**
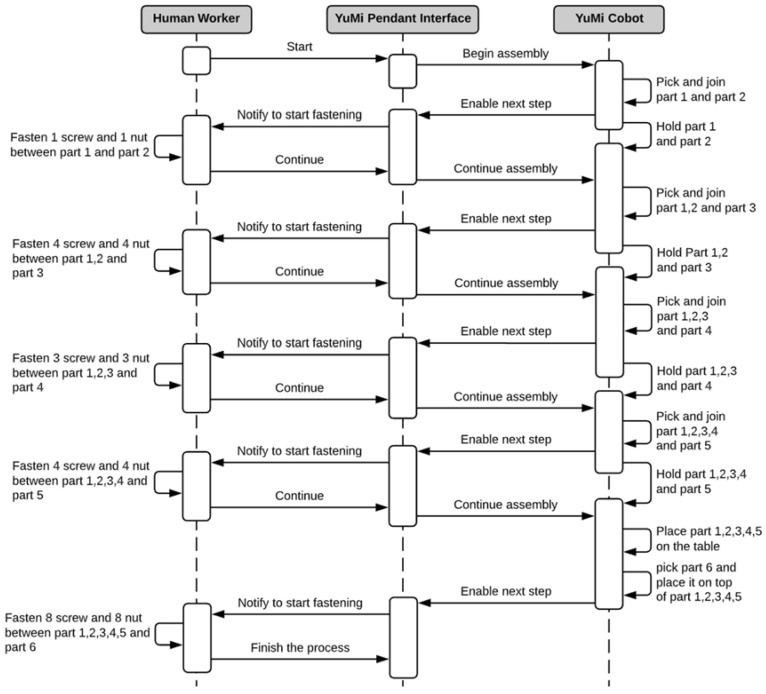
Sequence diagram of the assembly process of the box.

The assembly process is depicted in Figure 8 as a sequence diagram. The process initially involves the human work, the cobot and the interface pendant for the communication. The sequence starts with human worker triggers the robot to pick the 1st and the 2nd faces and hold them together. Once done, the robot will notify the user to start the fastening process. The user then starts the fastening task while the robot is holding the parts. These two tasks are executed in parallel. 

Then, the human notifies the cobot to continue the process by joining the next part. This process is repeated for the 3rd, 4th, and 5th part in the box. The difference is the number of the joining screws. In addition, the manipulation of the box by the robot is different based on the fixated sides of the box. Once the robot and the human assemble the 5th side of the box. The cobot places the box on the working table and then joins the 6th part on top of the previously assembled part to form the closed box. After that, the human installs the remaining screws to finish the assembly. It is important to mention that the human will be performing additional tasks besides screwing. In fact, the human worker will also help in wiggling the side to reach complete fit. Furthermore, the human worker will be preparing the screws and nut while the robot is working on the box. This highlights the need to trustworthy feeling as the human will not be seeing the robot and to trust that the robot is performing correctly.

### 4.2. Data Gathering and Human Profiling

As described in Section 3.2, the human profiling process requires collecting data for the user with different stimulus for estimating the range of emotions during the operational mode. In this regard, and for this research, the EmotivPro software was used for extracting the emotions of the user as metrics. These metrics are scaled particularly for each user on a 0% to 100% scale. Meaning that for the same stimulus, depending on the personal characteristics of an individual, two users could register different values for the same metric. Additionally, due to the software’s algorithms, the more times the headset is used it will generate more data, and in return, the metrics will be more precise. To leverage from this aspect, the training and experiments have been performed on one user incrementing the data and developing a more accurate emotional profile. Thus, three tests have been conducted for the data collection. These tests include valence-arousal test, box assembly alone test and box assembly with Yumi test.

**Valence-Arousal Test**: Inspired by the Valence-Arousal emotions classification method, this experiment consisted of monitoring how emotional metrics vary as the subject was exposed to different videos. The content was chosen to evoke alterations in Interest, Excitement, Focus and Relaxation. Considering the description of the metrics, Interest represents Valence and Excitement reflects Arousal. The experiment was divided into three parts. Each part focuses on inducing some specific levels of emotions. To assess the veracity of the metrics, the subject was asked to evaluate the feelings regarding the target emotions in a range of four levels: low, mid-low, mid-high and high. Later, the values monitored by the headset will be contrasted with the subject auto evaluation. 

In the first part, the subject was exposed to various videos which tried to induce a relaxation feeling, meaning, low Excitement levels and middle-Interest values. The second set of videos was selected to evoke high Valence-Arousal emotions in the user. Finally, the third group has as intention increasing the degree of aversion feelings on the subject. Divided into two subsets of videos, the first one target sadness and the second one stress and anger. 

**Figure 9:**
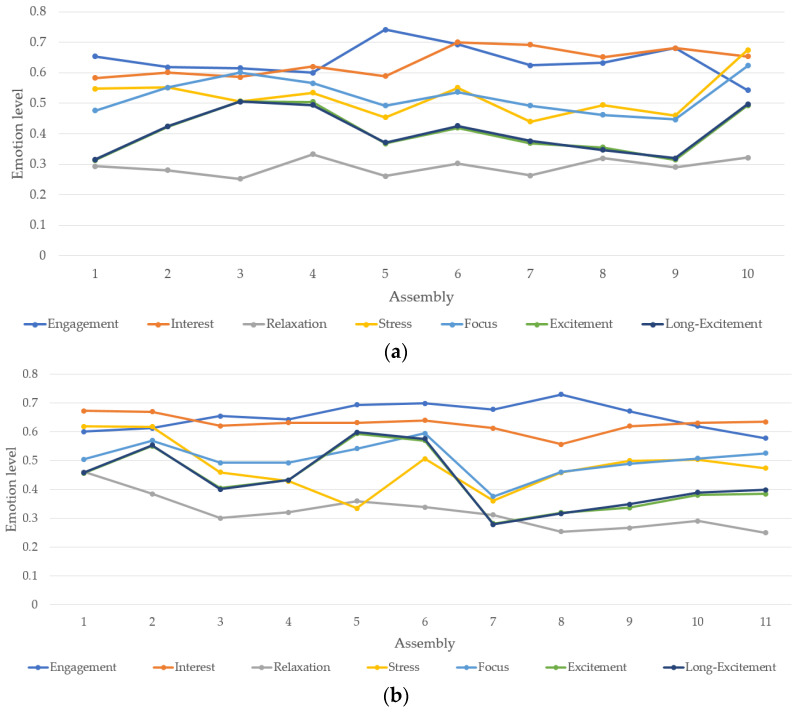

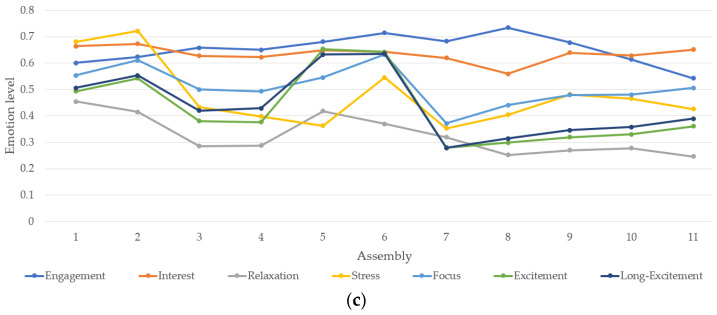
Average values of human profiling process: (**a**) box assembly alone, (**b**) box assembly with the cobot including the waiting time for the cobot, (**c**) box assembly with the cobot excluding the waiting time for the cobot.

**Box assembly alone**: In this experiment, the subject had to fulfil the task of assembling the wooden box by himself. This test aims at providing a reference of the humans’ emotions during the assembly process. Furthermore, by comparing the results with the ones recorded working with YuMi it would be possible to extract better conclusions about handling the task alone or in a collaborative way with a cobot. The box was assembled ten times by the user, following the same order of steps. See Figure 9a. Once the ten assemblies were finished, it was calculated maximum, minimum and average values for all the emotion metrics. Taking into consideration these values, it is possible to analyses if there is progress on the mental state and if the user is improving his abilities to fulfil the task producing a reduction of negative emotions as stress. 

**Box assembly with the cobot**: This last experiment consisted of assembling the wooden box with the collaboration of YuMi. In this case, the assembly of the box was performed eleven times, following the same steps, and calculating maximum, minimum and average values of each emotional metric in order to make comparisons with the previous experiment. In addition, it is important to mention that the data for this experiment has been calculated with two different considerations. Firstly, taking into consideration the whole time of the assembly composed by the user’s working time and the user’s waiting time between steps as depicted in Figure 9b. Secondly, just considering the working time of the user as shown in Figure 9c. This difference has been taken to compare properly the assembly without YuMi with the assembly with YuMi.

Looking on the collected data, the following observation can be identified:

- The Stress average value is lower working with YuMi than working alone. The reason might be related to the fact of sharing responsibilities in the work field with the cobot. As the assembly process is very stressful to be conducted with only two hands.
- Engagement emotion is higher working with the cobot than alone. This could represent that the user is more entertained performing the task in a collaborative way.
- Focus metric results to be lower with YuMi than assembling the box alone. This can be cause by the fact of sharing task with the robot, and then, waiting the robot to finish the task.

As mentioned before, in order to make a better analysis of the data, it could be more meaningful extracting the idle time while working with the cobot. In this regard, and after comparing the performance of the user without including the idle time, the following observation can be identified:

- The Stress metric, even being higher than considering the idle time, remains lower than assembling the box without assistance. This fact confirms that the subject is less stressed collaborating with the cobot than making the task alone.
- Engagement barely changes subtracting idle time, being higher collaborating with YuMi than alone.
- Focus increases as expected by not considering the idle time, being closer to the values working alone. Lower values of Focus might reflect that the subject needs less concentration effort to fulfil the task.

**Figure 10:**
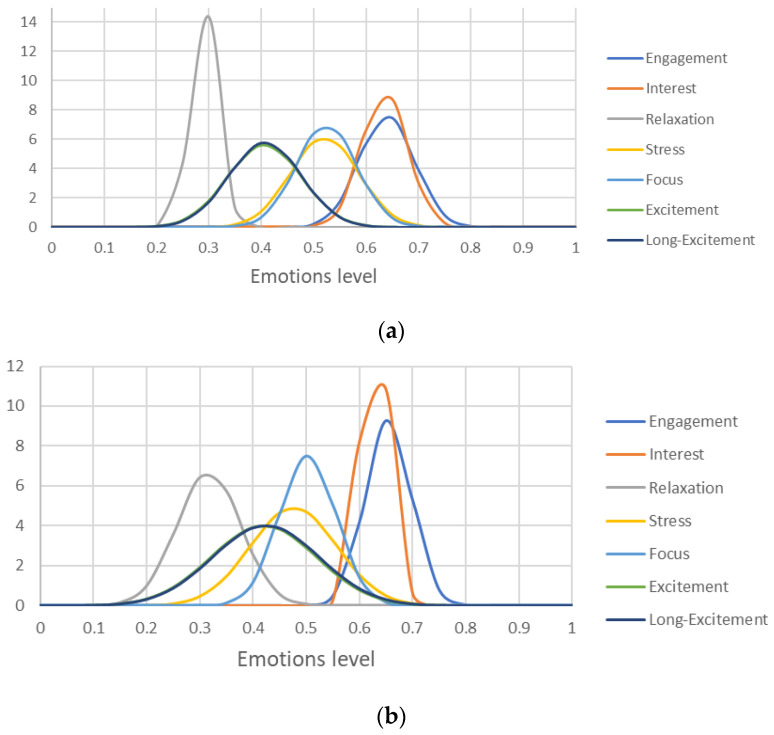

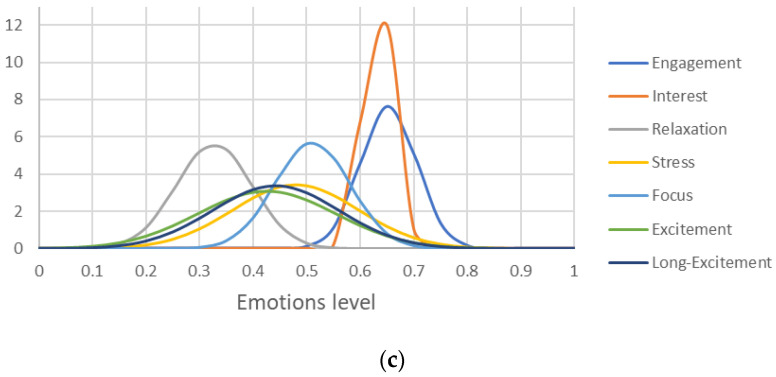
Normal distributions of the human profiling data: (**a**) box assembly alone, (**b**) box assembly with the cobot including the waiting time for the cobot, (**c**) box assembly with the cobot excluding the waiting time for the cobot.

Furthermore, and in order to ease the analysis of the data, normal distributions have been created for each test for the emotions as depicted in Figure 10. Following the figures, it is important to mention that, for these experiments, Excitement and Long-excitement metrics seems to have a close correlation with each other. Moreover, Stress also reflects the relaxation state of the user. In other words, being less relaxed translates to being more stressed and vice-versa. Consequently, Excitement and Interest have a lower relevance in this situation. In addition, the stress emotion reflected the most variance when changing the tests conditions. moreover, interest and engagement show a noticeable correlation. As a conclusion based on the collected data, the most relevant metrics to be evaluated for this application are the Stress, which showed noticeable changes in the mean values and variance; Engagement and Focus emotions of the human worker which both showed independence form Stress and each other. Finally, Table 2 presents the mean and standard deviation of the normal distributions of the stress, engagement focus emotions. 

### 4.3. Operational Mode and the Rule Configuration

Following the architecture that is presented in Section 3.2, the first component which needs to be developed is the EEG DataStream Adapter. This component translates the signals of the EEG to emotions. As the Epoc+ headset has been utilized for this research, the EmotivPro application provides the needed requirement. It provides the aforementioned 7 emotions in Table 1 based on the signals of the 14 EEG electrodes. Then, the next component to be developed is the Rules Engine and more specifically the Rules themselves. In this regard, and by using Emotiv’s API, the rule engine receives the emotion metrics in a JSON array every ten seconds. The application saves the incoming data and calculates an average for every emotion type. Considering the average values and utilizing Stress as a principal metric and Engagement and Focus as complementary, this component compares the data with predefined ranges of values for the mentioned metrics. These ranges are extracted from the profiling tests and defined using five levels of classification: LOW, MID-LOW, MID, MID-HIGH and HIGH. The actual metrics of these levels are dependent on the human worker reactions. For the robot, and for the research purposes, two parameters have been selected for representing the robot adaptation. These parameters comprise of velocity and delay. The velocity parameter reflects the velocity at which the cobot is moving. Meanwhile, the delay reflects the time that the cobot waits in a position where the user can fasten the screws. In relation to the human emotions, the robot velocity is proportional with the engagement and the focus and inversely proportional with the stress. On the contrary, the delay parameter is proportional to the stress and inversely proportional to the engagement and the focus. These relations can be observed the logic rules.

The design of the rules is mainly done using trial and error approach and is inspired by the process of tuning of the rules in the fuzzy controllers. A dominant emotion must be selected for such approach as an initial emotion. In this research, the stress emotion has been considered as the dominant emotion following the outcome of the profiling process as presented previously in Figure 9 and Figure 10 and Table 2. Once the dominant emotion is selected, the initial rules are created considering only the stress emotion. As an example, and as the stress is an inversely proportional with robot speed, the initial rules consider FAST speed for the robot if the stress is LOW or MID_LOW, MID speed if the stress is MID, and SLOW speed for MID_HIGH and HIGH stress. These initial rules can be seen in rule 1, 3, 7, 8 and 11 if the engagement and the focus emotions are ignored. Once these rules are stated, the fine-tuning process starts by introducing one emotion at a time to see the effect on the speed. This process led to create rule 2 from rule 1, rule 4 from rule 3, rule 5 and 6 from rule 7, rule 9 from rule 8, and rule 10 from rule 11. Finally, the fine tuning also can propagate to cover the segmentation of the emotions. In this context, the segmentation refers to the converting process form numeric to states. By default, the states are divided linearly considering the minimum and maximum recorded emotions in the profiling process as the upper and lower edges of the range. Then, a proper adjustment can be introduced. The overall goal is creating representative relations of the robot speed and the human’s emotions.

(1) low(str) ∧ ¬(mid_low(eng) ∨ low(eng)) ∧ ¬(mid_low(foc) ∨ low(foc))→fast(vel)

(2) low(str) ∧ (mid_low(eng) ∨ low(eng)) ∧ (mid_low(foc) ∨ low(foc))→mid(vel)

(3) mid_low(str) ∧ ¬(low(eng)) ∧ ¬(mid_low(foc) ∨ low(foc))→fast(vel)

(4) mid_low(str) ∧ ¬(low(eng)) ∧ (mid_low(foc) ∨ low(foc))→mid(vel)

(5) mid(str) ∧ mid_low(eng) ∧ (mid(foc) ∨ mid_high(foc) ∨ high(foc))→fast(vel)

(6) mid(str) ∧ (mid_high(eng) ∨ high(eng)) ∨ mid_low(foc)→fast(vel)

(7) mid(str) ∧ ¬(mid_low(eng) ∨ low(eng)) ∧ low(foc)→mid(vel)

(8) mid_high(str)∧(mid_low(eng)∨low(eng))∧(mid(foc)∨mid_low(foc)∨low(foc))→slow(vel)

(9) mid_high(str)∧(mid(eng)∨ mid_high(eng)∨high(eng))∧(mid_high(foc)∨high(foc))→slow(vel)

(10) high(str) ∧ (mid_high(eng) ∨ high(eng)) ∧ (mid_high(foc) ∨ high(foc))→mid(vel)

(11) high(str) ∧ ¬(mid_high(eng) ∨ high(eng)) ∧ ¬(mid_high(foc) ∨ high(foc))→slow(vel)

(12) fast(vel)→short(del)

(13) mid(vel)→mid(del)

(14) slow(vel)→long(del)

Once the rules have been identified and configured, the Robot Adapter and robot application can be programmed. The Robot Adapter establishes the communication with the cobot. Accordingly, this component listens to the incoming messages from the cobot and sending the parameters’ information regarding the user’s emotional state. In addition, a TCP client socket is created and a communication loop with the cobot is maintained. Depending on the received message from the cobot, it emits signals to initiate or terminate the emotional feedback, thus knowing if the cobot is starting or ending and step. Finally, the robot is hosting the TCP server which receives the messages form the Robot Adapter, and accordingly, changes the speed and the delay in the robot program following the aforementioned rules.

## 5. Results and Discussion

To demonstrate the approach, the assembly of the box shown in Figure 5b is conducted 5 times where the same operator and robot conducted the test. During each assembly process, the emotions have been recorded and monitored while the robot is adapting to the human emotions. Table 3 shows the overall measurements and parameters of the experiment. The experiment flowed the sequence diagram in the Figure 8 where the human worker is expected to perform 5 tasks per assembly process for fastening the screws. For this experiment the last task is excluded as it ends with the human performing the closure of the box without the help of the robot. 

**Figure 11:**
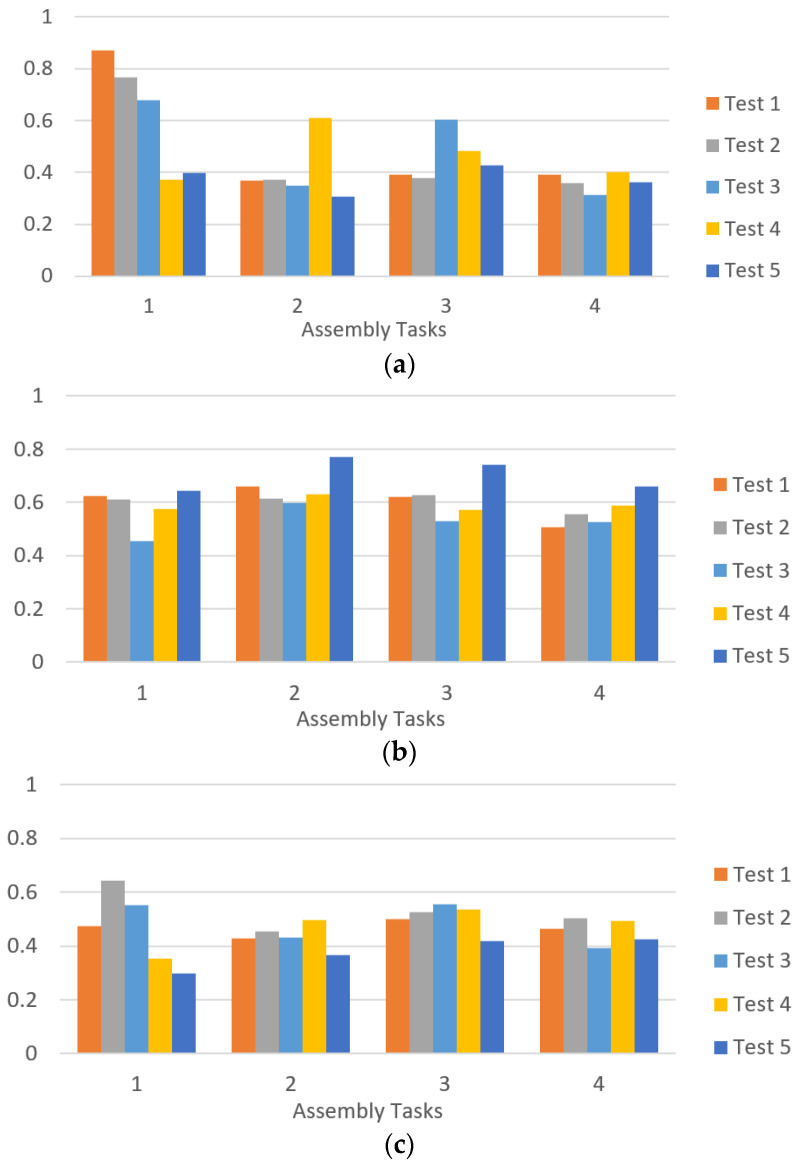
Emotion’s level of the human worker during the 5 tests: (**a**) stress level; (**b**) engagement level; (**c**) focus level.

Figure 11a depicts the stress level of the worker during the tests. It is possible to observe that the human in the first moment was highly stressed while performing the first step. As a result, the cobot reduced its velocity and increased the delay so the user can have more time to fasten the bolts. Consequently, the stress level drops down. Afterwards, and while performing the 2nd task, the speed is increased to FAST again due to the low stress level during this task. Due to complications in the step 3, the stress is increased causing the cobot to change its speed to MID. The reduction on the velocity and the increase of the delay makes the user reduce its stress to a low level. The last task presents stable level of stress throughout the 5 tests. It is obvious that the worker had sudden stress increase in the stress level in task 2 during the 4th test and task 3 during the 3rd and the 4th tests. These changes occurred due to dropped screws from the hands of the worker.

For the engagement and the focus in Figure 11b,c respectively, the levels where stable through the tests. It is observed that the focus level increased slightly once a stressful moment happened. This can be seen in the focus levels in the 2nd and the 3rd tasks after the stress increased. For the engagement, a slight decay is observed in the levels as the human worker repeats the tasks which is an indication of less interest in performing the tasks. For the robot velocity, it is observed that as the tests continued, the speed reached stable values as FAST. However, the speed was unstable in the 1st and the 3rd tests. This observation indicates that an adjustment is required on the rules to avoid instability in the change of the robot’s velocity. In addition, the ranges and the amount of the states can also be increased to reach better resolution in the emotions’ recognition.

Overall and on another remark, the selection of the robot’s parameters in this research was leaning towards the demonstration of the research rather than solving a problem in a real use case. Thus, for generalizing the approach of selecting the parameters of the robots. The authors suggest first understanding the relations between the robot’s parameters and the human’s emotions. As an example, a test can be added in the profiling stage to understand the reaction to the human while varying the robot’s parameters. Then the selection of the robot parameters can be conducted by selecting the most effective parameters that are related to the problem.

In relation with the previous work, this research does not deviate from the facts that are listed in [60], which considers the EEG technology as a solution for training the users, enhancing the interaction with the robots and providing better assessment of the users. In fact, this research provides deeper insights on the challenges and the advantages of emotion-based application for controlling the robots as it increases the trustworthy between human and robots. Nonetheless, and aligned with the research in [61], the current technology may still present a limitation of such approach as the measurement of the emotions can be inaccurate and suffers from low levels of repeatability.

## 6. Conclusions

The presented approach demonstrated the exploitation of the emotions of the human worker in a human-robot collaboration environment. This exploitation reflected the effect of the trust and comfort levels of the human during a collaborative work. As robots and cobots lack of humans’ emotional recognition, this research can be helpful and beneficial for developing emotional-drive solution for adjusting the collaborative robots. The conducted literature revealed that among facial, speech, body, Electrocardiogram (ECG) and Electroencephalography (EEG) human emotions recognition method, the latter demonstrated to be the most accurate and reliable technique for digitizing the human emotions. Nonetheless, the EEG is a haptic sensing method which can be uncomfortable, and sometimes unapplicable to be used in a realistic use case. For the developed approach, a commercially available headset and an in-house made headset are used to digitize the human’s emotions. Then, an application is developed to demonstrate the cobot adaptability using the emotions of a human worker. Additionally, a first-order rule-based engine is used to segment sensed emotions and adjust the robot’s parameters. As a result, the more time spent in repeating the task, the more comfortable the worker become. On the contrary of that, the engagement can be decrease once the wok becomes more a routine to the worker. For the focus, the level can be increase as the human is under stress with contrite to the mistakes. 

For future work, the approach can be enhanced in considering higher resolution in the segmentation which can reduce the effect of the positive and negative feedback. In addition, developing the rules can elaborated to understand the internal effect of the emotions. In other words, during experimentations, the stress has internal effect on both the engagement and the focus. This internal effect creates a semi-dependent system which can be studied more in the future to void instability in the robot reactions. Finally, other human’s body signals like ECG and EMG can be considered for future work to provide redundant and accurate emotions’ analysis.

## Figures and Tables

**Table 1:** Description of the emotions that are measured using Epoc+ EEG headset.

Emotion	Description
Interest	Level of appeal or repulsion to a stimulus
Stress	Grade of serenity or pleasure induced by a situation
Relaxation	Ability to reach a tranquil mental state
Excitement	Degree of immediate stimulation generated by an action
Engagement	State of immersion in the implemented activity
Long-term Excitement	Analogous to the excitement metric but measured in longer time intervals, such as minutes
Focus	Capacity to continuously concentrate on a particular task while eluding distractions

**Table 2:** Mean and std. deviation values for the human profiling data of the selected emotions (Stress, Engagement and Focus).

	Box Assembly Alone			Box Assembly with the Cobot Including the Waiting Time for the Cobot			Box Assembly with the Cobot Excluding the Waiting Time for the Cobot		
	Engagement	Stress	Focus	Engagement	Stress	Focus	Engagement	Stress	Focus
Mean	0.6401	0.5214	0.5247	0.6543	0.4763	0.5027	0.6528	0.4795	0.5107
Std. Deviation	0.0523	0.0650	0.0570	0.0429	0.0814	0.0530	0.0521	0.1172	0.0703

**Table 3:** Cobot parameters based on the stress, engagement, and the focus levels during the 5 tests.

Test	Assembly Task	Stress	Engagement	Focus	Velocity	Delay
1	1	HIGH	MID-LOW	MID-LOW	SLOW	LONG
	2	MID-LOW	MID-LOW	MID-LOW	FAST	SHORT
	3	MID-LOW	MID-LOW	MID-LOW	FAST	SHORT
	4	MID-LOW	LOW	MID-LOW	MID	MID
2	1	MID-HIGH	MID-LOW	MID	SLOW	LONG
	2	MID-LOW	MID-LOW	MID-LOW	FAST	SHORT
	3	MID-LOW	MID-LOW	MID	FAST	SHORT
	4	MID-LOW	MID-LOW	MID-LOW	FAST	SHORT
3	1	MID-HIGH	MID-LOW	MID	SLOW	LONG
	2	MID-LOW	MID-LOW	MID-LOW	FAST	SHORT
	3	MID	MID-LOW	MID	MID	MID
	4	MID-LOW	MID-LOW	MID-LOW	FAST	SHORT
4	1	MID-LOW	MID-LOW	MID-LOW	FAST	SHORT
	2	MID	MID-LOW	MID-LOW	MID	MID
	3	MID-LOW	MID-LOW	MID	FAST	SHORT
	4	MID-LOW	MID-LOW	MID-LOW	FAST	SHORT
5	1	MID-LOW	MID-LOW	LOW	FAST	SHORT
	2	MID-LOW	MID	MID-LOW	FAST	SHORT
	3	MID-LOW	MID	MID-LOW	FAST	SHORT
	4	MID-LOW	MID-LOW	MID-LOW	FAST	SHORT
